# PAH-specific therapy for pulmonary hypertension and interstitial lung disease: A systemic review and meta-analysis

**DOI:** 10.3389/fcvm.2022.992879

**Published:** 2022-11-17

**Authors:** Ning Zhao, Jun Chen, Mingming Zhang, Lihui Zhou, Lisong Liu, Jie Yuan, Xingxue Pang, Dayi Hu, Xiaoxia Ren, Zhongyi Jin

**Affiliations:** ^1^Department of Geriatrics, Chui Yang Liu Hospital Affiliated to Tsinghua University, Beijing, China; ^2^Capital Medical University, Beijing, China; ^3^Department of Cardiology, Chui Yang Liu Hospital Affiliated to Tsinghua University, Beijing, China; ^4^Cardiac Rehabilitation Center, Dongzhimen Hospital, Beijing University of Chinese Medicine, Beijing, China; ^5^Department of Cardiovascular Medicine, Dongzhimen Hospital, Beijing University of Chinese Medicine, Beijing, China; ^6^Department of Cardiology, Peking University People's Hospital, Beijing, China

**Keywords:** pulmonary hypertension, interstitial lung disease, 6-min walk distance (6MWD), treprostinil, pulmonary arterial hypertension (PAH)

## Abstract

**Objective:**

Pulmonary hypertension (PH) in context with interstitial lung disease (ILD) portends serious clinical consequences and a high rate of mortality. Recently published randomized controlled trials (RCTs) which assessed the pulmonary arterial hypertension (PAH)-specific drugs for pulmonary hypertension and interstitial lung disease (PH-ILD) revealed inconsistent clinical outcomes with previous studies. We conducted a systemic review and meta-analysis to further investigate the effect of PAH-specific therapies for PH-ILD.

**Methods:**

Clinical trials were searched from the EMBASE, PUBMED, and CENTRAL databases. The duration from the establishment of the database to June 2022 for RCTs evaluates the effect of PAH-specific therapy in patients with PH-ILD. RevMan 5.4 was used for the meta-analysis.

**Results:**

A total of six articles (with a total of 791 patients) were included, including 412 patients in the treated group and 379 patients in the control group. As compared to placebo, the change of 6MWD was a significant improvement with PAH-specific therapy in the six RCTs (23.09; 95% CI, 12.07–34.12 *P* < 0.0001); but when the study with inhaled treprostinil was excluded, the significant improvement in the change of 6MWD from baseline was not present anymore (MD 11.01, 95%CI−6.43–28.46 *P* = 0.22). There was no significant improvement in the change in lung function, hemodynamic parameters, clinical worsening, all-cause death, and serious adverse effects in the treated group compared to placebo.

**Conclusion:**

PAH-specific therapy significantly improved exercise capacity in the patients with PH-ILD, but this is due to the greater contribution of the study with inhaled treprostinil. Therefore, our findings still did not support the routine use of the whole PAH-specific drugs for PH-ILD.

## Introduction

Interstitial lung disease (ILD) is a group of clinical disorders characterized by different degrees of inflammation and fibrosis in the lung interstitial tissues, such as idiopathic pulmonary fibrosis (IPF), and idiopathic interstitial pneumonia (IIP). The subtype and stage of ILD was the main influence factor and it was associated with IPF severity, 8–83% of patients with idiopathic pulmonary fibrosis (IPF) progressively develop PH, which resulted in lower exercise capacity, decreased quality of life, greater need for supplemental oxygen, and higher mortality compared to ILD alone (1, 2). Precapillary pulmonary hypertension is defined as an elevation in mean pulmonary arterial pressure and pulmonary vascular resistance (3). In the World Health Organization (WHO) classification of pulmonary hypertension, precapillary pulmonary hypertension due to lung disease is classified as group 3 defined by pulmonary artery wedge pressure (PAWP) ≤15 mmHg and mean pulmonary arterial pressure (mPAP ≥ 25 mmHg) (4) in contrast to Group 1 PH, where intima and media remodeling predominate, pulmonary vascular remodeling is the main contributor to Group 3 PH, which is mainly characterized by changes in the media.

Current pulmonary arterial hypertension (PAH) specific drugs have demonstrated efficacy and safety in a Group 1 PAH patient population, which were divided into three classifications due to three pathogenesis of PAH and pharmacological characteristics, including endothelin receptor antagonists (ERA), phosphodiesterase type 5 inhibitors (PDE5-i), prostacyclin pathway. PAH-specific therapies induced pulmonary vasodilation and showed anti-proliferative activities on the pulmonary vasculature, reducing pulmonary vascular resistance and ultimately right ventricular (RV) afterload in PAH, whereas these vasodilating drugs with oral administration may result in ventilation/perfusion (V/Q) mismatch, especially in the setting of significant oxygen deficit (5). Although, some pilot studies with ERA and PDE5-I have previously shown improvement in hemodynamics and no deleterious effect on gas exchange for Group 3 PH patients. Conversely, more clinical trials with rigorous study designs were inconsistent and failed to show a significant clinical benefit in various ILD or PH-ILD patients, although those drugs were effective in treatment with PAH (6–8). Recently published articles with inhaled vasodilating drugs have shown a significant improvement in exercise capacity for patients with PH-ILD (9, 10). To gain better insight, we conducted this systematic review and meta-analysis to evaluate the whole PAH-specific drugs on exercise capacity and lung function in PH-ILD.

## Methods

This systematic review and meta-analysis were conducted in accordance with the Preferred Reporting Items for Systematic reviews and Meta-Analyses (PRISMA) guidelines (11).

### Search strategy and selection criteria

A systematic literature search was performed in electronic databases, including PubMed, EMBASE, and the Cochrane Central Register of Controlled Trials (CENTRAL) from inception to June 5, 2022. The main search terms were pulmonary hypertension, interstitial lung disease (ILD), idiopathic interstitial pneumonia (IIP), idiopathic pulmonary fibrosis (IPF), endothelin receptor antagonists (ERA), phosphodiesterase type 5 inhibitors (PDE5-i), and prostacyclin in addition to associated Clinical Trials filter. We only included studies published in English. A detailed search strategy for literature was presented in Supplementary Table 1. We searched PROSPERO for similar clinical designs to avoid duplication. We also searched ClinicalTrials.gov to identify other studies and according to the reference lists of included studies, a backward and forward snowballing approach was used to retrieve relevant literature.

Inclusion criteria were as follows: (1) the study population included patients >18 years old with PH or borderline PH (group 3) as defined in the 2015 ESC/ERS Guidelines and interstitial lung disease including IPF, IIP (4). (2) Any randomized controlled clinical studies of evaluation of efficacy and safety in these patients with PAH-specific drugs, (3) reported exercise capacity such as change of 6-min walk distance (6MWD) from baseline, (4) reported at least one of the following outcomes: changes in lung function, hemodynamic assessment, (5) reported the rate of hospitalization at the end of the study.

Exclusion criteria were as follows: the patients in the controlled arm of RCTs who take other PAH-specific drugs were excluded. Single-arm prospective studies, retrospective studies, observational studies, review articles, letters, and case reports were excluded. Additional exclusion criteria were a crossover design, assessment of clinical effects, patients with pulmonary hypertension other than group 3, RCTs without treatment effects, and pregnancy.

### Data extraction and outcome measures

Firstly, we designed an excel form to extract data on basic characteristics and primary outcomes. Two investigators (JC and ZYJ) reviewed the full text and extracted the following data from each included article. The primary outcomes were as follows: (1) Exercise capacity includes the 6-min walk stance (6MWD), and brain natriuretic peptide (BNP) levels. (2) Hemodynamic parameters include the change in mean pulmonary artery pressure (mPAP), pulmonary vascular resistance (PVR), cardiac index (CI) or cardiac output (CO), and right atrial pressure (RAP) measured by right heart catheterization (RHC) both at baseline and follow-up time points. (3) Lung function includes the change of diffusing capacity of carbon monoxide (DLCO) and forced vital capacity (FVC). (4) Clinical worsening or disease progression, all-cause death, serious adverse effects (SAE).

### Quality assessment

Assessment of the risk of bias was conducted by the Cochrane Collaboration risk for bias tool with two independent reviewers identifying the studies (NZ and LHZ). For the ARTEMIS-PH study, risk of bias analysis was conducted based on the information from www.clinicaltrials.gov (NCT00879229) and from the results of the published ARTEMIS study (12, 13) which presented the trial design and results of ARTEMIS-PH in brief.

### Sensitivity analysis

The sensitivity analysis investigated whether a single study affected the overall results of the combination, which would have an impact on comprehensive research in the following two situations. First, when a study is deleted, the result will be significantly different. If there is little difference in the overall results when a study is deleted, it indicates the sensitivity of the combined results, and the results obtained are unstable. Second, the results show sensitivity and stability, and the conclusion is correct.

### Statistical analysis

The mean difference (MD) and their respective 95% confidence intervals (CI) were calculated based on a fixed-effect model (FED) of the inverse variance estimation method. The standardized mean difference (SMD) was used as an effective measure of continuous data when studies assessed the same endpoint with different ways of measurement. Statistical heterogeneity testing was analyzed by Cochrane's Q statistic and *I*^2^ statistic. *I*^2^ values >25, 50, and 75% were considered evidence of low, moderate, and high statistical heterogeneity, respectively. If *I*^2^ values were >50%, the pooled analysis was calculated based on a random-effects model (REM). *P*-values were two-tailed and an alpha level of 0.05 was considered a statically significant difference. Meta-analyses were conducted using Review Manager version 5.4 (RevMan; The Cochrane Collaboration, Oxford, UK).

## Results

### Search results

**Figure 1:**
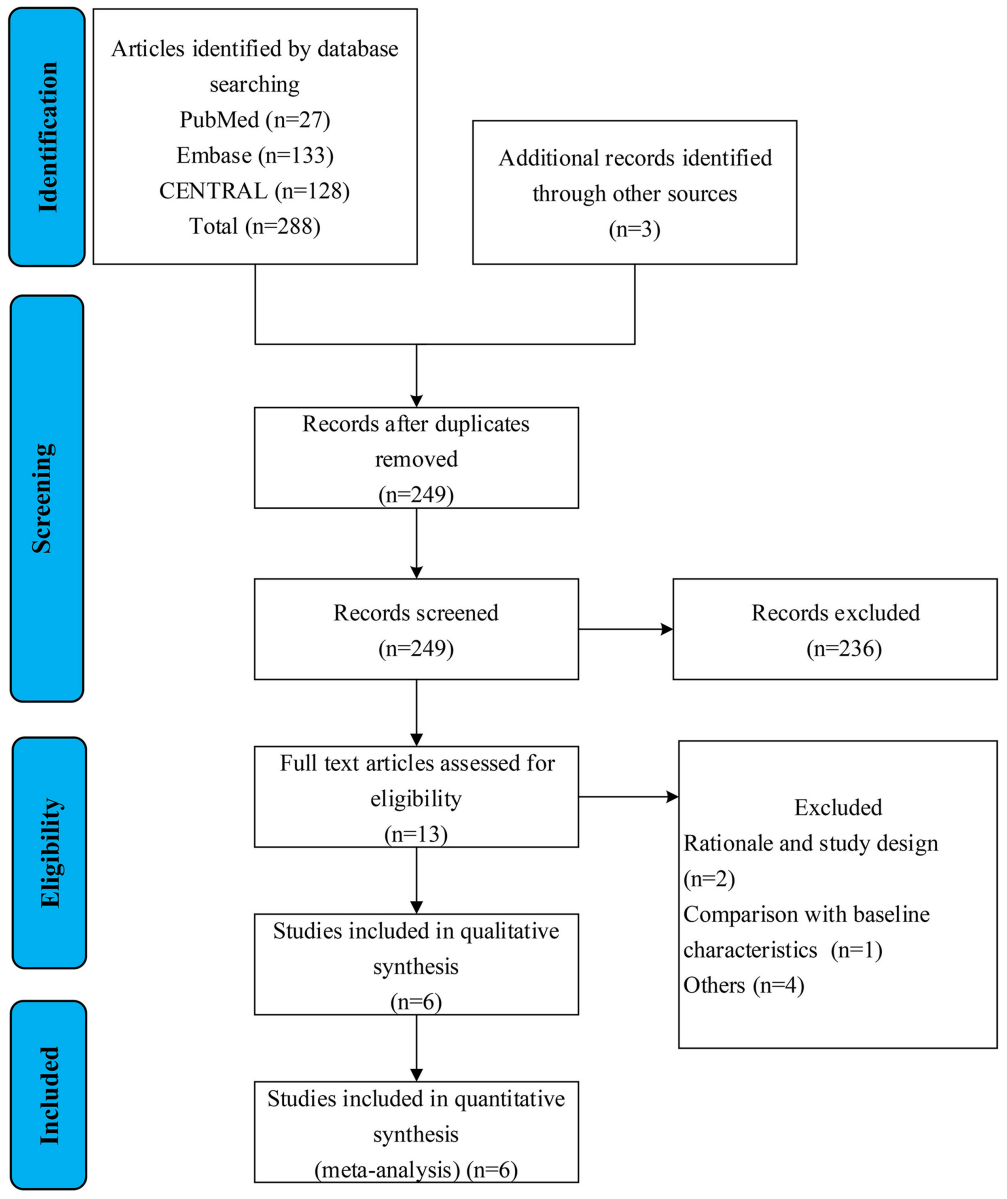
Selection flow chart of literature screening.

Search results and reasons for exclusion were listed in the literature screen flow diagram (Figure 1). A total of six RCTs that evaluate the efficacy and safety between treated patients and placebo were finally included in our meta-analysis (9, 10, 13–16). One article was identified from www.clinicaltrials.gov (NCT00879229). Four articles did not meet the prespecified research target including that two articles, which were early excluded due to not meeting significant clinical benefits and serious adverse effects (Table 1).

**Table 1:** Characteristics of included studies.

**Study**	**Study type**	**Blinding**	**Duration** **(weeks)**	**PH due to** **lung disease**	**N patients** **(intervention/** **control)**	**Intervention**	**Dose**	**Endpoints**	**Results**
Corte et al. (15) (BPHIT)	Multicenter RCTs	Double-blind	16 weeks	Fibrotic IIP	60 (40/20)	Bosentan	125 mg bid	mPAP, PVRi, CI, RAP, BNP, 6MWD, QoL, FVC, SpO2, DLCO	Negative
ARTEMIS-PH (13)	Multicenter RCTs	Double-blind	16 weeks	IPF	40 (25/15)	Ambrisentan	10 mg daily	6MWD, WHO FC, FVC	Negative
Nathan et al. (16)	Multicenter RCTs	Double-blind	26 weeks	IIP	147 (73/74)	Riociguat	0.25–0.5 mg tid	6MWD, FVC, WHO FC, Time to clinical worsening, blood gas analysis	Negative
Behr et al. (14)	Multicenter RCTs	Double-blind	52 weeks	IPF	177 (88/89)	Sildenafil	20 mg tid	6MWD, Disease progression, FVC, FVC1, all-cause mortality	Negative
Nathan et al. (9)	Multicenter RCTs	Double-blind	8 weeks	Fibrotic ILD	41 (23/18)	Inhaled NO	30 mg/kg ideal body weight/h	Physical activity parameters, 6MWD, oxygen saturation	Positive
Waxman et al. (10)	Multicenter RCTs	Double-blind	16 weeks	ILD	326 (163/163)	Inhaled treprostinil	Nebulizer in up to 12 breaths (total, 72 μg) four times daily	6MWD, NT-proBNP, Time to clinical worsening	Positive

RCTs, randomized controlled trials; IIP, idiopathic interstitial pneumonia; IPF, idiopathic pulmonary fibrosis; ILD, interstitial lung disease; NO, nitric oxide; 6MWD, 6-min walk distance; mPAP, mean pulmonary artery pressure; PVR, pulmonary vascular resistance; PVRi, pulmonary vascular resistance index; CI, cardiac index; BNP, brain natriuretic peptide; QoL, quality of life; NT-proBNP, N-terminal pro–B-type natriuretic peptide; SpO2, oxygen saturation measured by pulse oximetry.

The included studies were conducted from 2014 to 2021. This meta-analysis included a total of 791 patients with PH-ILD. A large proportion (41.2%) of patients were drawn from the INCREASING trial (*n* = 326) (Table 1).

### Quality assessment

**Figure 2:**
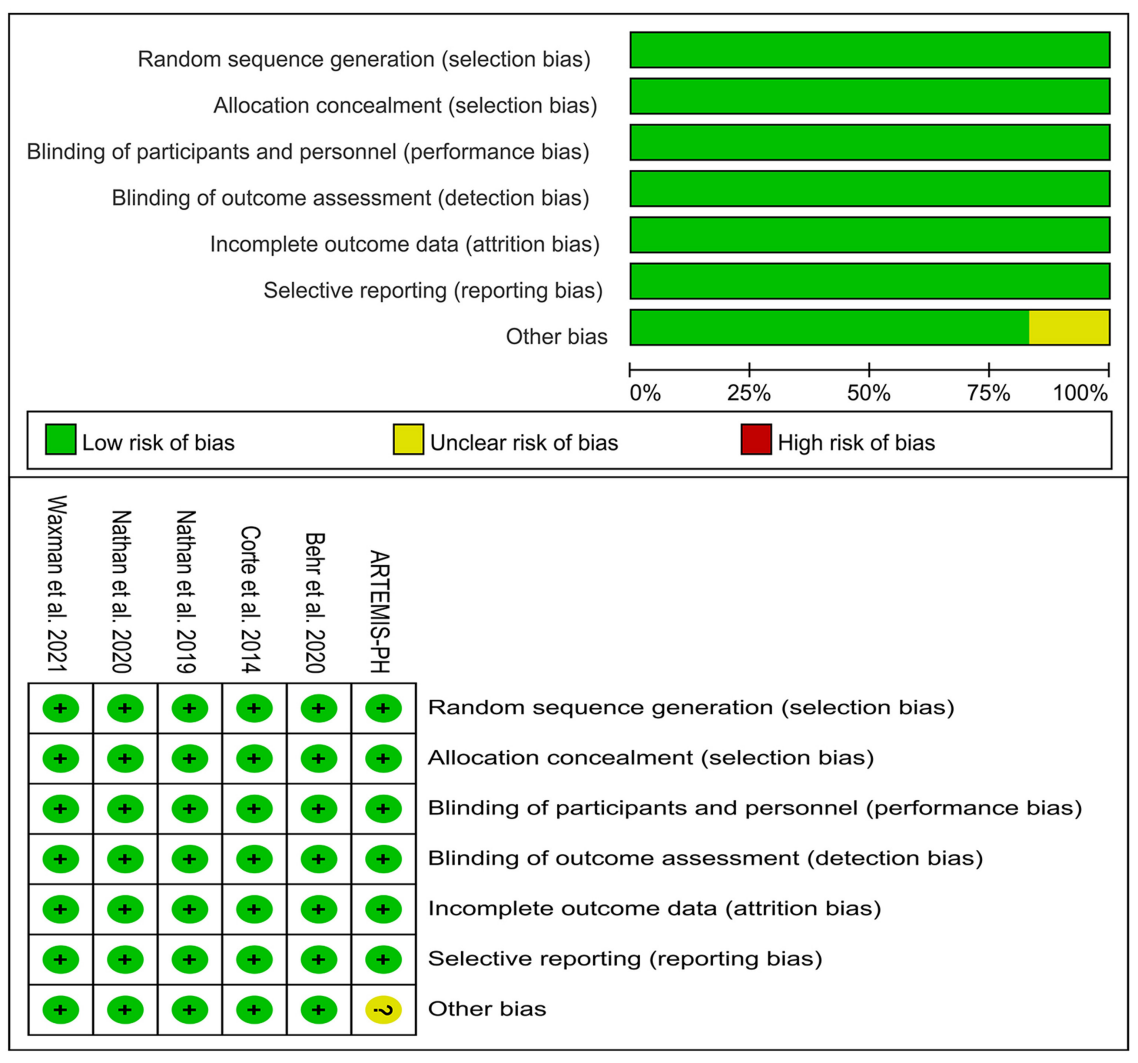
Risk of bias assessment of included studies.

We conducted a risk of bias analysis of all included studies to evaluate the overall quality of the studies. For all included RCTs, there was a lower risk of bias. There was an increased risk of bias in the ARTEMIS-PH due to the early termination of the trial (Figure 2).

### Exercise capacity

**Figure 3:**
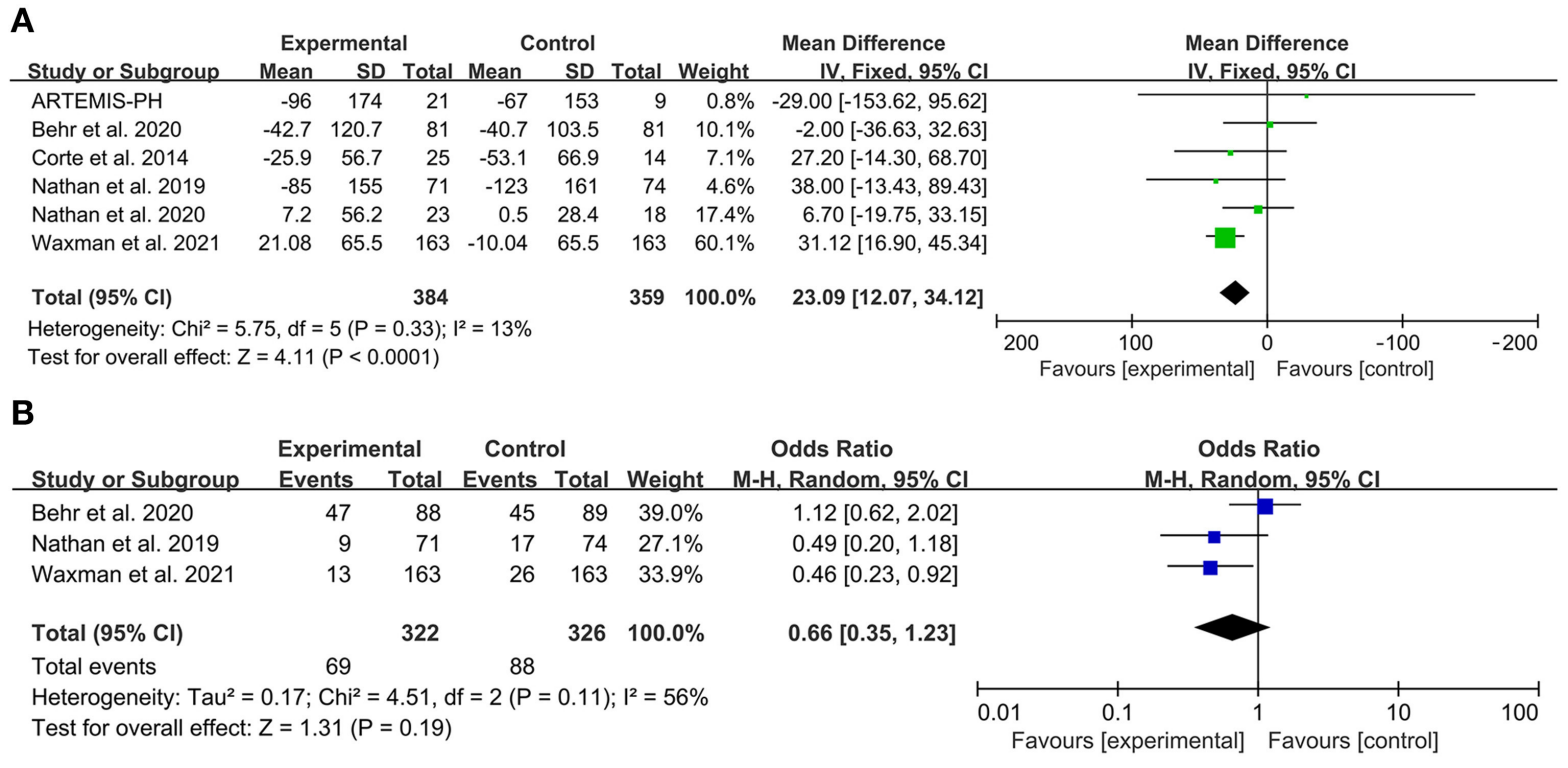
Forest plot illustrating a comparison of effects of PAH-specific therapy on exercise capacity in PH-ILD. **(A)** Change of 6MWD from baseline. **(B)** Decline of 6MWD from baseline >15%.

Six articles reported the mean change of 6MWD from baseline that was significantly improved with PAH-specific therapy compared to placebo. The FEM was then used for analysis. The meta-analysis results showed that MD: 23.09; 95% confidence interval [CI], 12.07–34.12 *P* < 0.0001 and there was no significant heterogeneity (*I*^2^ = 13% *P* = 0.33) in these six studies. For the sensitivity analysis, when the study by Waxman et al. was excluded, the significant improvement in the change of 6MWD from baseline was not present (MD 11.01, 95% CI−6.43–28.46 *P* = 0.22).There was no significant heterogeneity (*I*^2^ = 0% *P* = 0.61) in the rest of five studies (Figure 3).

Three articles reported the decline in 6MWD > 15% from baseline. The heterogeneity test showed that *I*^2^ = 56% and *P* = 0.11, which indicated that there was moderate heterogeneity in the literature. The REM was then used for analysis. The meta-analysis results showed that OR: 0.66, 95% CI: 0.35 to 1.23, Z = 1.31, and *P* = 0.19 (Figure 3).

### Lung function

**Figure 4:**
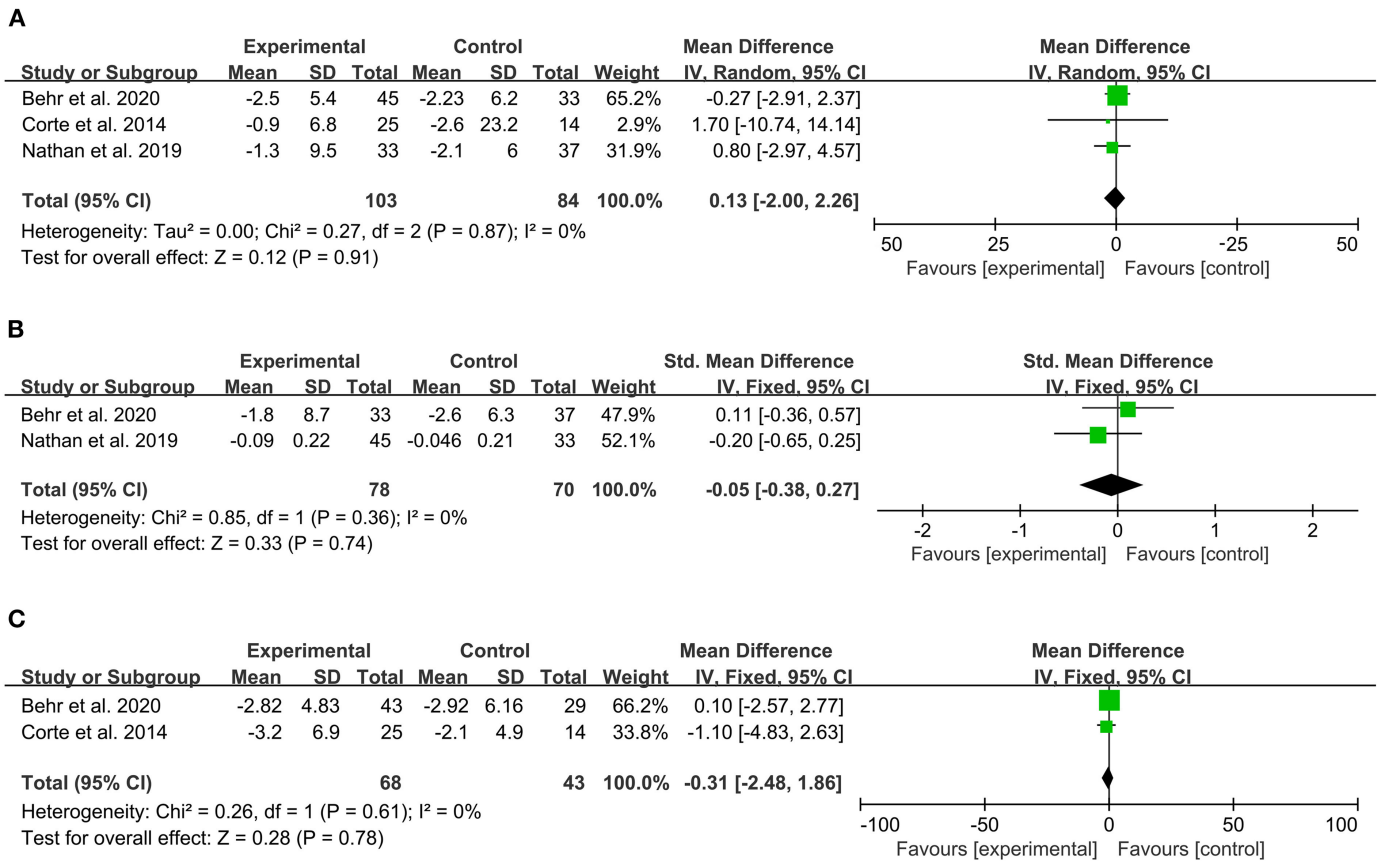
Forest plot illustrating a comparison of effects of PAH-specific therapy on lung function in PH-ILD. **(A)** Change of FVC, % predicted. **(B)** FEV1, % predicted **(C)** DLCO, % predicted.

Three articles reported change of FVC predicted from baseline, which did not significantly improve with PAH-specific therapy compared to placebo. The heterogeneity test showed that *I*^2^ = 0% and *P* = 0.87, which indicated that there was no significant heterogeneity in the literature. The FEM was then used for analysis. The meta-analysis results showed that MD: 0.13, 95% CI:−2–2.26, Z = 0.12, and *P* = 0.91 (Figure 4).

Two articles reported the change of FEV_1_ predicted from baseline. The heterogeneity test showed that *I*^2^ = 0% and *P* = 0.36, which indicated that there was no significant heterogeneity in the literature. The FEM was then used for analysis. The meta-analysis results showed that SMD:−0.05, 95% CI:−0.38–0.27, Z = 0.33, and *P* = 0.74 (Figure 4).

Two articles reported the change of DLCO predicted from baseline. The heterogeneity test showed that *I*^2^ = 0% and *P* = 0.61, which indicated that there was no significant heterogeneity in the literature. The FEM was then used for analysis. The meta-analysis results showed that MD:−0.31, 95% CI:−2.48–1.86, Z = 0.28, and *P* = 0.78 (Figure 4).

### Serious adverse effect

**Figure 5:**
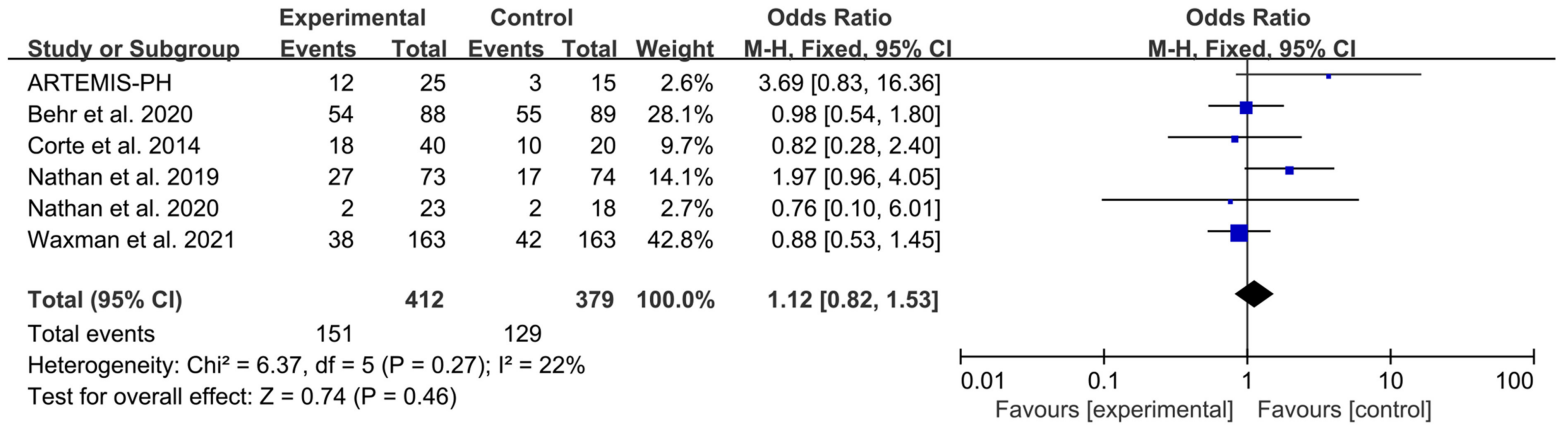
Forest plot illustrating a comparison of any SAE in PH-ILD.

Six articles reported the patient number of any SAE. The heterogeneity test showed that *I*^2^ = 22% and *P* = 0.27, which indicated that there was no significant heterogeneity in the literature. The FEM was then used for analysis. The meta-analysis results showed that OR: 1.12, 95% CI: 0.82–1.53, Z = 0.74, and *P* = 0.46. The forest plot is shown in Figure 5.

Hemodynamics assessment, clinical worsening or disease progression, all-cause death (see the Supplementary materials, Supplementary Figures 1–3).

## Discussion

The present systematic review and meta-analysis firstly assessed the efficacy and safety of PAH-specific drugs in patients with PH-ILD. Interesting, there was a significant improvement in exercise capacity in the treatment group compared to the placebo. However, there was no significant difference in lung function, hemodynamic parameters, disease progression or clinical worsening, all-cause death, and SAE between the treatment group and placebo. Overall, our findings still did not support that the whole PAH-specific therapy was effective in routine clinical management with PH-ILD. In addition, inhaled administration of treprostinil or nitric oxide (NO) provided a new medication strategy and effective clinical results for patients with PH-ILD.

Although there was a significant improvement in a change of 6MWD from baseline with PAH-specific therapy in the patients with ILD-PH, it still does not validate the routine use of PAH-specific therapy in these patients due to the following reasons. First, we noted RCTs on ERAs have failed to show improvement in exercise capacity or hemodynamic parameters. In the BPHIT trial, bosentan did not effectively improve exercise capacity or reduce symptoms (15). Also, the ARTEMIS-PH trials of ambrisentan, an endothelin receptor antagonist, were terminated early due to increased SAE (13). Second, The RISE-IIP trial of riociguat, a soluble guanylate cyclase (sGC) stimulator, even showed harmful effects in PH-ILD populations; the RISE-IIP study was consequently terminated early due to increased rates of SAE and mortality; it also showed no significant improvement in 6MWD and banned from treatment in this indication (16). Third, sildenafil combined with pirfenidone also did not provide clinically meaningful benefits compared to the placebo, thus it was not an appropriate treatment for these patients (14). In the above included four RCTs, the exercise capacity in change of 6MWD from baseline was negative in both the treatment group and placebo. It was well known that vasodilating effect due to differential action pathway of these PAH-specific drugs, one primary cause for systemic administration of vasodilating drugs might increase or aggravate V/Q mismatch and shunt, resulting in worsening hypoxemia and wasting of the small ventilatory reserve of these patients (17). In addition, vascular reactivity was fundamentally different in pulmonary vascular bed located in otherwise normal lung tissue cemented in fibrotic tissue in PH-ILD (18, 19). The reason that the change of 6MWD from baseline in our meta-analysis was positive is mainly the contribution of the other two positive studies with inhalative administration with treprostinil or nitric oxide. Through sensitivity analysis in included articles, we found that the significant improvement in results of the change of 6MWD did not remain by excluding the INCREASING study, and it revealed this study was a large weight coefficient in our pooled analysis and the result of significant improvement in the change of 6MWD was unstable. Inhalative administration of vasodilating drugs results in optimizing V/Q matching, which will be distributed preferentially to the well-ventilated areas of the lungs as well as reduced the distribution of vasodilating drugs in the poor-ventilated areas. This point of view has been raised based on previous studies with single-dose iloprost, and the results showed an improvement in hemodynamics and exercise capacity (19, 20). The consistent therapeutic pathway, inhaled nitric oxide also has shown the potential effects to improve oxygenation in patients with fILD, which selective dilated the pulmonary vasculature in well-ventilated areas of the lung, but these positive single-arm studies usually observed acute responses and did not design a placebo group (21, 22). The recent RCTs such as the iNO-PF study with a small sample size have further proved this effect and inhaled NO facilitated improvement in physical activity, moreover it was safe and well-tolerated (9). To date, The INCREASING study with the largest patient population for inhaled treprostinil treated in PH-ILD showed a positive outcome with a 31-meter placebo-corrected improvement in the primary endpoint of 6MWD (10). Positive FVC trends in *post-hoc* analysis contributed to improvements in the 6-min walk distance, reflecting both interstitial and vascular changes (23). In addition, treatment with inhaled treprostinil was associated with a lower risk of clinical worsening and relatively fewer adverse effects. Inhaled treprostinil was firstly approved by the US FDA for the indication in the treatment of PH-ILD due to the results of the above clinical studies.

Treprostinil is a tricyclic benzidine analog of epoprostenol with chemical stability, which inhibited platelet aggregation, acted as an antiproliferative effect, and direct dilated the pulmonary vascular bed. moreover, these studies that inhalative administration with prostacyclin or prostacyclin analog for PH-ILD have shown significant improvement in exercise capacity, but not in intravenous systemic administration or oral administration. Thus, the inhalative administration was a promising treatment approach for patients with PH-ILD, as the inhaled route resulted in a high local concentration in the best ventilated areas, ultimately reducing V/Q mismatch.

Although a previous study has shown that PAH-specific therapies slightly improved mPAP and PVR in group 3 PH, our results were inconsistent because only two RCTs were included for pooled analysis (24). Few previous studies demonstrated a positive effect on FVC in treatment with PAH-specific therapy for PH-ILD and our finding was consistent with it (18). In the aspect of all-cause death and SAE, no significant difference was observed in the results of pooled analysis. The RISE-IIP study reported increased rates of SAE and mortality in the riociguat group, leading to early termination. In another study, serious treatment-high rates of emergent adverse events (61 vs. 62%) were reported in patients in the sildenafil group and the placebo. Moreover, a high rate of mortality (22 vs.26%) was also reported in the two groups (16). Conversely, the INCREASING study and iNOPF study reported a relatively lower rate of adverse events (9, 10).

Therefore, we still did not ensure that the whole class of PAH-specific drugs is effective for PH-ILD, despite inhalative administration with treprostinil or nitric oxide, which was an effective safe, well-tolerated and novel treatment approach.

## Limitation

Limitations of the study should be acknowledged. First, the number of included studies and sample size of the meta-analysis was small and the treatment duration was relatively short. Second, we only included English language articles and thus we could have missed an article written in other languages. Third, the PH diagnosis in the included studies was defined by RHC or echocardiography, thus it increased the relative risk of bias in the included population.

## Conclusion

In conclusion, PH in context with ILD portends serious clinical consequences and a high rate of mortality. PAH-specific therapy significantly improved exercise capacity in the patients with PH-ILD due to the contribution of the studies with inhalative administration with treprostinil and nitric oxide. However, our findings still did not support the routine use of the whole PAH-specific therapy in these patients, despite inhaled treprostinil being recommending for PH-ILD.

## Data availability statement

The original contributions presented in the study are included in the article/Supplementary material, further inquiries can be directed to the corresponding author/s.

## Author contributions

Conception and design: DH, ZJ, and JC. Implementation, collection, analysis, and interpretation of data: NZ, XR, XP, MZ, LZ, JY, LL, ZJ, and JC. Drafting of the manuscript or revising it critically for important intellectual content: JC, NZ, ZJ, and XR. Final approval of the manuscript submitted: ZJ and XR. All authors contributed to the article and approved the submitted version.

## Conflict of interest

The authors declare that the research was conducted in the absence of any commercial or financial relationships that could be construed as a potential conflict of interest.

## Publisher's note

All claims expressed in this article are solely those of the authors and do not necessarily represent those of their affiliated organizations, or those of the publisher, the editors and the reviewers. Any product that may be evaluated in this article, or claim that may be made by its manufacturer, is not guaranteed or endorsed by the publisher.

## Abbreviations

PAH: pulmonary arterial hypertension
PH: pulmonary hypertension
ILD: interstitial lung disease
IIP: idiopathic interstitial pneumonia
IPF: idiopathic pulmonary fibrosis
ERA: endothelin receptor antagonists
PDE5-i: phosphodiesterase type 5 inhibitors
iNO: inhaled nitric oxide
6MWD: 6-min walk distance
FVC: forced vital capacity
FEV_1_: forced expiratory volume in 1 s
DLCO: diffusing capacity of the lung for carbon monoxide
SAE: serious adverse effect.
