# Supplementary material for: PAH-specific therapy for pulmonary hypertension and interstitial lung disease: A systemic review and meta-analysis
Source: Front Cardiovasc Med. 2022 Nov 17;9:992879. doi: 10.3389/fcvm.2022.992879 (PMC9713234; doi:10.3389/fcvm.2022.992879)
Supplement: Supplementary file 1 [file Table_1.DOCX]

Supplementary table

| PUBMED | #1 "pulmonary hypertension"[Title/Abstract] | 41437 |
| --- | --- | --- |
|  | #2 "interstitial lung disease"[Title/Abstract] OR "pulmonary fibrosis"[Title/Abstract] OR "interstitial pneumonia"[Title/Abstract] | 8852 |
|  | #3 "Sildenafil"[Title/Abstract] OR "tadalafil"[Title/Abstract] OR "bosentan"[Title/Abstract] OR "ambrisentan"[Title/Abstract] OR "macitentan"[Title/Abstract] OR "iloprost"[Title/Abstract] OR "epoprostenol"[Title/Abstract] OR "treprostinil"[Title/Abstract] OR "nitric oxide"[Title/Abstract] OR "riociguat"[Title/Abstract] OR "Selexipag"[Title/Abstract] | 173077 |
|  | #4 #1AND #2 AND #3 | 151 |
|  | #5 #4AND (clinicaltrial[Filter]) | 27 |
| EMBASE | #1 'pulmonary hypertension'/exp OR 'pulmonary hypertension' OR (pulmonary AND ('hypertension'/exp OR hypertension)) | 159549 |
|  | #2 'interstitial lung disease'/exp OR 'interstitial lung disease' OR (interstitial AND ('lung'/exp OR lung) AND ('disease'/exp OR disease)) OR 'pulmonary fibrosis':ab,ti OR 'interstitial pneumonia':ab,ti | 139142 |
|  | #3 'sildenafil'/exp OR sildenafil OR tadalafil:ab,ti OR bosentan:ab,ti OR ambrisentan:ab,ti OR macitentan:ab,ti OR iloprost:ab,ti OR epoprostenol:ab,ti OR treprostinil:ab,ti OR 'nitric oxide':ab,ti OR riociguat:ab,ti OR selexipag:ab,ti | 227802 |
|  | #4 #1AND#2AND#3 | 1033 |
|  | #5 #4 AND 'clinical trial'/de | 133 |
| CENTRAL | #1 (pulmonary hypertension):ti,ab,kw | 5641 |
|  | #2 (interstitial lung disease):ti,ab,kw OR (pulmonary fibrosis):ti,ab,kw OR (interstitial pneumonia):ti,ab,kw | 5284 |
|  | #3 #1 AND #2 | 347 |
|  | #4 (epoprostenol):ti,ab,kw OR (treprostinil):ti,ab,kw OR ("nitric oxide"):ti,ab,kw OR (riociguat):ti,ab,kw OR (selexipag):ti,ab,kw | 9400 |
|  | #5 ("sildenafil"):ti,ab,kw OR (tadalafil):ti,ab,kw OR (bosentan):ti,ab,kw OR (ambrisentan):ti,ab,kw OR (macitentan):ti,ab,kw | 3721 |
|  | #6 (iloprost):ti,ab,kw | 502 |
|  | #7 #4 OR #5 OR #6 | 13165 |
|  | #8 #3 AND #7 | 128 |
