# Supplementary material for: PAH-specific therapy for pulmonary hypertension and interstitial lung disease: A systemic review and meta-analysis
Source: Front Cardiovasc Med. 2022 Nov 17;9:992879. doi: 10.3389/fcvm.2022.992879 (PMC9713234; doi:10.3389/fcvm.2022.992879)
Supplement: Supplementary file 2 [file Data_Sheet_1.DOCX]

**Supplementary materials**

**1. Efficacy outcomes**

**Hemodynamic assessment**

Two articles reported the change of mPAP from baseline. The heterogeneity test showed that I^2^=0% and P=0.42, which indicated that there was no significant heterogeneity in the literature. The FEM was then used for analysis. The meta-analysis results showed that MD: -2.39, 95% CI: -6.27 to 1.49, Z= 1.21, and P=0.23. The forest plot is shown in supplementary figure 1.

Two articles reported the change of PVR or PVRi from baseline. The heterogeneity test showed that I^2^=0% and P=0.63, which indicated that there was no significant heterogeneity in the literature. The FEM was then used for analysis. The meta-analysis results showed that SMD: -0.56, 95% CI: -1.1 to -0.02, Z= 2.05, and P=0.04. The forest plot is shown in supplementary figure 1.

**Clinical worsening or disease progression**

Five articles reported the clinical worsening or disease progression. The heterogeneity test showed that I^2^=0% and P=0.49, which indicated that there was no significant heterogeneity in the literature. The FEM was then used for analysis. The meta-analysis results showed that OR: 0.77, 95% CI: 0.56 to 1.07, Z= 1.55, and P=0.12. The forest plot is shown in supplementary figure 2.

**All-cause death**

six articles reported the all-cause death. The heterogeneity test showed that I^2^=15% and P=0.32, which indicated that there was no significant heterogeneity in the literature. The FEM was then used for analysis. The meta-analysis results showed that OR: 0.98, 95% CI: 0.59 to 1.63, Z= 0.09, and P=0.93. The forest plot is shown in supplementary figure 3.

**2. Figure legends**

Supplementary figure 1. Forest plot illustrating a comparison of hemodynamic parameters in PH-ILD (a) mPAP (b) PVR or PVRi

Supplementary figure 2. Forest plot illustrating a comparison of clinical worsening or disease progression in PH-ILD.

Supplementary figure 3. Forest plot illustrating a comparison of all-cause death in PH-ILD.

**3. Egger’s test and funnel plot in the change of 6MWD from baseline**

**
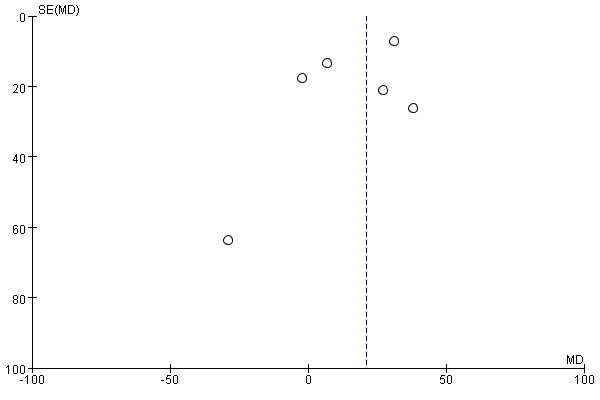
**
