# Supplementary figures and images for: PAH-specific therapy for pulmonary hypertension and interstitial lung disease: A systemic review and meta-analysis
Source: Front Cardiovasc Med. 2022 Nov 17;9:992879. doi: 10.3389/fcvm.2022.992879 (PMC9713234; doi:10.3389/fcvm.2022.992879)

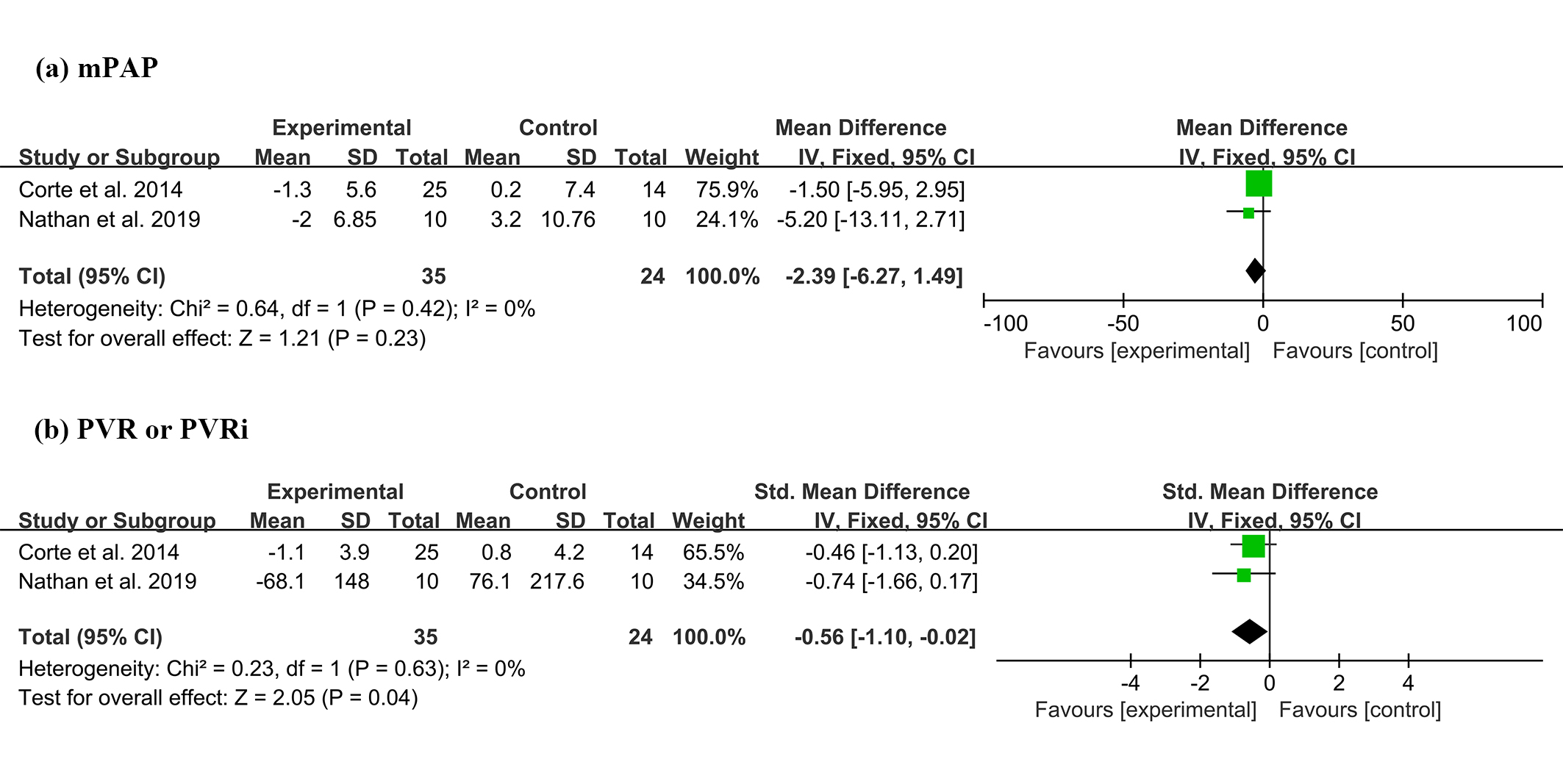

Supplement: Supplementary file 3 [file Image_1.JPEG]

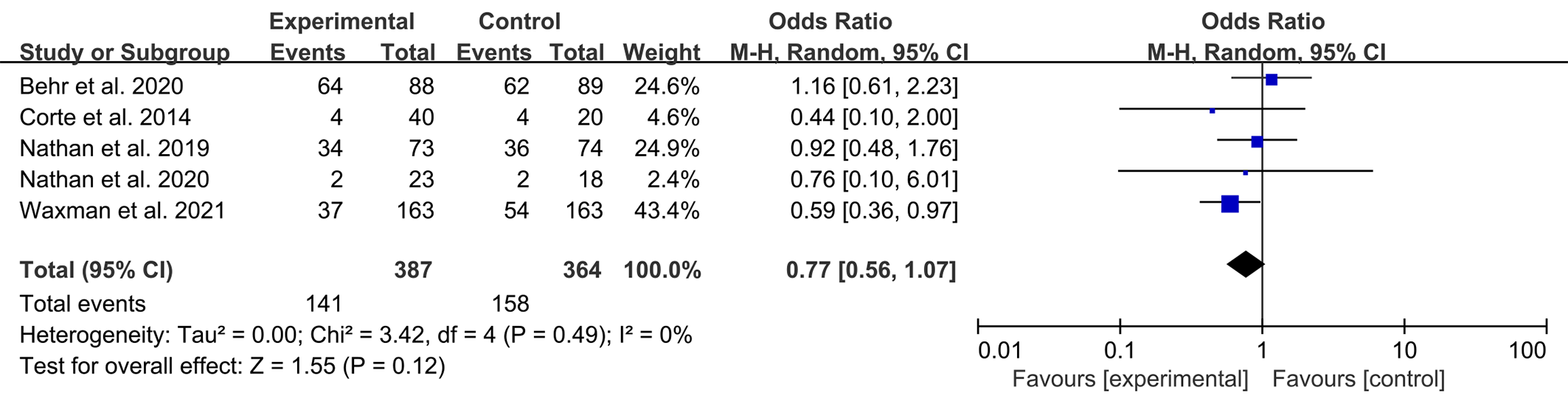

Supplement: Supplementary file 4 [file Image_2.TIF]

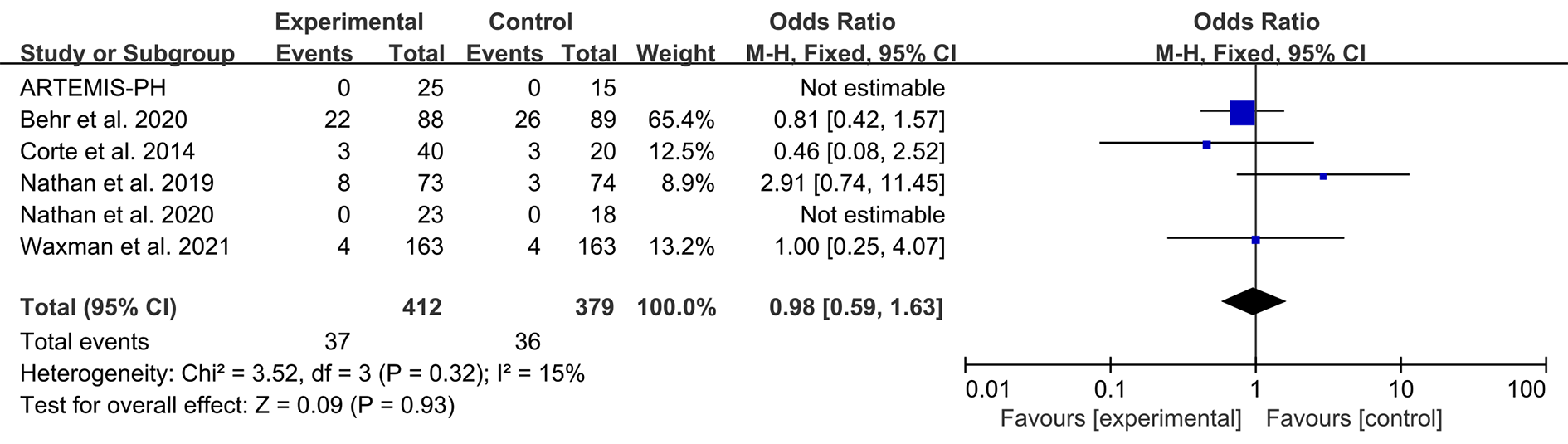

Supplement: Supplementary file 5 [file Image_3.TIF]
